# Application of Patient-Specific Instrumentation in a Dog Model with Antebrachial Growth Deformity Using a 3-D Phantom Bone Model

**DOI:** 10.3390/vetsci9040157

**Published:** 2022-03-25

**Authors:** Hee-Ryung Lee, Gareeballah Osman Adam, Shang-Jin Kim

**Affiliations:** 1 Hansarang Animal Hospital, Seoul 02880, Korea; hr-lee@hanmail.net; 2 Department of Veterinary Medicine and Surgery, College of Veterinary Medicine, Sudan University of Science and Technology, Khartoum P.O. Box 204, Sudan; gorba000@gmail.com; 3 R&D Division, HUVET Co., Ltd., Iksan 54531, Korea; 4 College of Veterinary Medicine, Jeonbuk National University, Specialized Campus, Iksan 54596, Korea

**Keywords:** antebrachial growth deformity, dog, osteotomy, patient-specific instrumentation, 3-D

## Abstract

One of the most frequent bone deformities in dogs is antebrachial growth deformity (AGD), which results from malunion of the distal growth plates. The objective of the present study was to re-align the limbs, which can correct the length mismatch and reset the coherence of the joint with the aid of a 3-D phantom model for surgical preplanning. A 14-month-old, intact female Golden Retriever with an angular deformity of the left radius and ulna was selected for the study. The diagnosis was confirmed by orthogonal radiographs. Moreover, computed tomography (CT) scans revealed a multiplane deformity with valgus, procurator, and external rotation of the left radius. The pre-surgical planning started with the quantification of the angular deformity, followed by a simulated virtual osteotomy, and concluded with an in vitro rehearsal surgery on 3-D printed phantom bone models. In the operating room, prefabricated patient-specific instrumentation (PSI) was attached at the planned site of the radial bone surface for a precise closing wedge osteotomy. Then two locking plates were fixed routinely. Post-operative radiographs showed accurate correction of the deformity as we had planned. At 12 weeks post-operatively, the follow-up surveys revealed improved gait, weight-bearing, and progression of bone healing. Our PSI design, based on novel surgical planning, was steady yet straightforward during the osteotomy. The osteotomy was performed without difficulty since the PSI that pre-determined the sites and angles let the surgeon perform the antebrachial malformation surgery. This method of operation reduces stress on the operator and helps to improve accuracy, repeatability, and surgery time.

## 1. Introduction

The disparity in growth between the radius and the ulna is associated with multiplanar contortions, which result in elbow joint deformity or inappropriate bodyweight stress on the carpal joint, as well as osteoarthritis in the elbow, carpal joints, or both [1]. In dogs, antebrachial growth deformities (AGDs) are the most common deformities, typically attributable to the extremes of chondrodystrophic formation or damaging premature closure of the distal growth plates [2,3,4,5,6,7].

Corrective osteotomy is advised if mobility and motion intolerance have changed, decreased, or secondary osteoarthritis of the carpal and elbow joints has progressed [3,4,5]. The purpose of corrective angular deformity osteotomy is to re-align the limbs, which can fix the length mismatch and reset the coherence of the joint. Corrective osteotomy requires a precise description of the features and range of deformation to plan open wedge osteotomy, closed wedge osteotomy, or dome osteotomy [4,6,7,8,9]. Correction can be immediate at the time of surgery or gradual with the use of external fixation incorporating hinged motors. Limb alignment using the external skeletal fixator has many advantages [10,11]. However, external skeletal fixation has the risk of pin-tract infection that is adversely related to morbidity compared to internal fixation [12,13].

Critical iatrogenic translational defects can result from applications of osteotomies that are inappropriate because of inadequately quantified bone deformities [14]. In the case of angular limb deformity corrective osteotomy, many studies have been conducted using 3-D techniques and rapid prototyping (RP) bone models. In particular, the improvement of the operation time, surgical invasion, surgical accuracy, patient pain, and patient-specific instrumentation (PSI) usefulness, such as risk, pre-operative planning, and surgical error reduction, have recently been demonstrated [15].

In general, programs that convert computed tomography (CT) data, which are used in many studies, into a 3-D printable file format (Stereo Lithography; STL; .stl) after bone segmentation and a 3-D surgical planning simulation program are expensive and require a professional workforce for the operation. Therefore, attempting to request 3-D print production of an affected bone model and patient-specific osteotomy guides from an external company may result in more than two weeks, several modifications, and relatively high costs [16,17]. Therefore, in this experiment, the PSI was designed for osteotomy saw blade guide and the bone plate and screw fixation with closing wedge corrective osteotomy.

In our previous report in the journal of animals [18], we demonstrated that the limitations of the freehand method could be overcome protocol in an in vitro environment using the PSI. Therefore, the goal of this study is to use the PSI protocol in the operating room on a dog with AGD. We reported the results and proposed novel methods based on successful findings.

## 2. Materials and Methods

### 2.1. Case Selection

**Figure 1:**
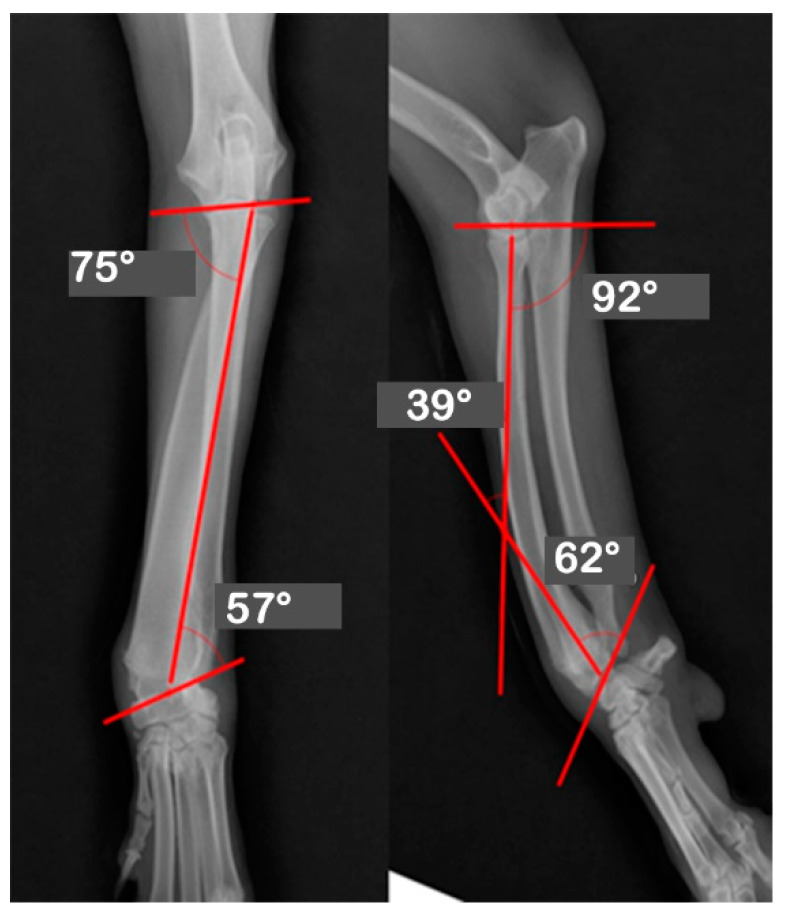
Orthogonal radiographs of the affected antebrachium. The anatomical angles of the proximal and distal radii were measured. The anatomic medial proximal radius angle (aMPRA) and the lateral distal radius angle (aLDRA) were initially 75° and 57°, respectively (Left). The anatomic caudal proximal radial angle (aCdPRA) measured 92°, while the caudal distal radial angles (aCdDRA) were 62°.

**Figure 2:**
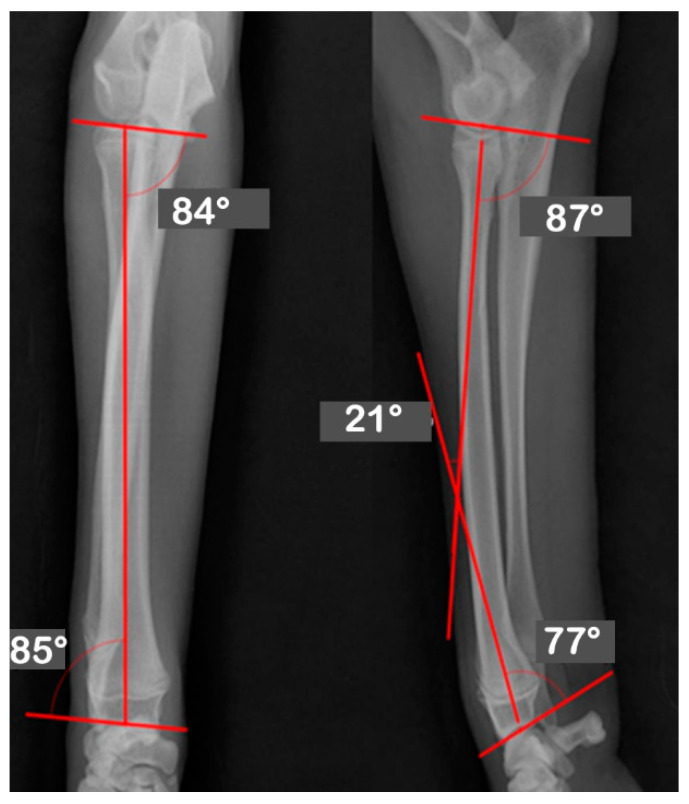
Orthogonal radiographs of the unaffected contralateral antebrachium. The anatomical angles of the proximal and distal radii were measured. The unaffected radius’s joint orientational angles, aMPRA, aLDRA, aCdPRA, and aCdDRA were 84°, 85°, 87°, and 77°, respectively.

At Hansarang animal clinic, a 14-month-old intact female Golden Retriever with an angular deformity of the left radius and ulna was treated with corrective osteotomy. Left forelimb lameness, restriction in flexion, radial shortening, and valgus of the left distal radius and ulna due to the partial premature closure of the epiphyseal growth plates were observed. A conventional orthogonal radiographic evaluation was performed. The anatomical angles of the proximal and distal radius were measured on frontal and sagittal view radiographs of the affected and unaffected contralateral radii. On the frontal view, the initial anatomical medial proximal radius angle (aMPRA; ref 82–83°; mean value 83°) and the lateral distal radius angle (aLDRA; ref 85–87°; mean value 86°) were 75° and 57°, respectively. On the sagittal view, the anatomical caudal proximal radial angle (aCdPRA; ref 84–86°; mean value 85°) measured value was 92°, while the caudal distal radial angle (aCdDRA; ref 76–78°; mean value 77°) measured value was 62° (Figure 1). The unaffected radius’s joint orientational angles, aMPRA, aLDRA, aCdPRA, and aCdDRA were 84°, 85°, 87°, and 77°, respectively (Figure 2). The magnitude of CORA for the case was 25° valgus and 30° procurvatum. The reference values were obtained from a previous study [19].

### 2.2. CT and Bone Image Segmentation

Stereolithographic 3-D triangular images of the radius and ulna of the affected and contralateral unaffected sides were generated by segmenting the bones from CT images (scanned with a 16-detector helical CT scanner (slice thickness, 0.7 mm; 120 kV; helical CT Alexion, TOSHIBA, Japan)) using 3-D slicer freeware (3-D Slicer, https://www.slicer.org/; version 4.8.0, accessed on 12 March 2021) [20,21,22]. The CT protocol for corrective osteotomies of the forearms included contiguous scanning of the whole forearm from the elbow to the radiocarpal joint. Manual thresholding and region growing were used for the bone image segmentation.

### 2.3. Preoperative Surgical Planning

The computer planning was performed on a desktop personal computer using the Windows 10 software application, 3-D builder (Microsoft Windows free application program, Microsoft Corporation, Redmond, WA, USA). The contralateral virtual bone model was mirrored and aligned with the pathological bone using the Iterative Closest Plane surface registration algorithm, to quantify the AGD in 3-D, as described in our published study [18]. A closing wedge osteotomy was planned [23]. To achieve a unique fit, PSI was designed to contain irregular convex and concave surfaces covering the bone from other directions, and for the corresponding surface of the guide to be placed on the bone as an exact replication of the surface of the bone model. A drill sleeve hole was designed for the most distal screw hole on the proximal fragment of the radius that can be drilled before the pre-contoured y-universal locking plate (YULP) is allowed to settle on the precise site. The PSI was fabricated in-house using the PMMA (Polymethyl methacrylate) material, based on the PLA model that was previously manufactured with the desktop 3-D printer device.

The authors chose 2.7 mm, a YULP, a compression locking plate (CLP), and locking screws (BS.COREM, Jeonbuk, Korea). After osteotomy simulation, the distal part was reduced, followed by stable plates that were pre-contoured based on the RP bone model surface in the planned correction. Cancellous bone or scaffold material for bone tissue engineering was not planned for this operation.

### 2.4. Pre-Medication and Anesthesia

Fluid therapy, 5% DW; antibiotics, Cefovecin 8 mg/kg SQ; analgesics, Butorphanol 0.3 mg/kg; Lidocaine, 0.5 mg/kg; and Ketamine, 0.5 mg/kg were mixed in one syringe. Meloxicam 0.2 mg/kg intravenous bolus injection was followed by Butorphanol 0.2 mg/kg/h (3.3 g/kg/min), Lidocaine 1.5 mg/kg/h (25 g/kg/min), and Ketamine 1.2 mg/kg/h (20 g/kg/min) added to 5% DW 100 mL bag; 50 mL/h constant rate of infusion. Ranitidine, 1 mg/kg, was subcutaneously injected as an antacid. General anesthesia was induced by Propofol 5 mg/kg IV followed by maintenance with 1–3 Vol% Isoflurane-oxygen. Lidocaine, 1 mL, was used to perform radial, ulnar, median, and musculocutaneous (RUMM) blocking anesthesia.

### 2.5. Surgery and Evaluation

As described in the previously published work [18], corrective osteotomies were preoperatively planned in 3-D, and patient-specific drills and cutting guides were designed based on the 3-D virtual simulation followed by fabrication. The final model of the PSI was applied intraoperatively to perform the closing wedge corrective osteotomy.

First, a lateral approach to the ulna was conducted for an ulnar osteotomy, with the approach made through a skin incision from the ulnar styloid to the midshaft. The radius was then reached cranially, with the skin incision extending from the radial diaphysis to the carpal joint.

**Figure 3:**
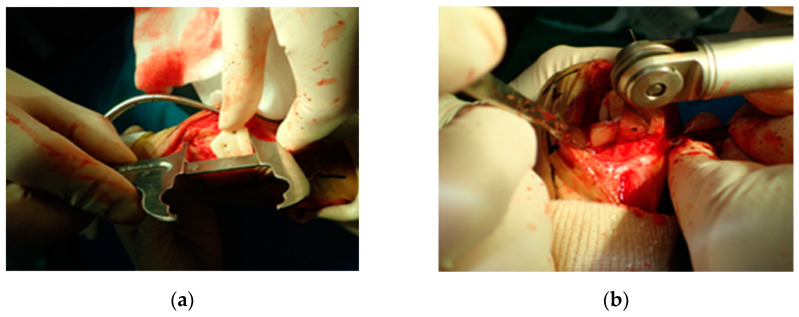
Distal patient-specific instrumentation (PSI) unit setting in situ using a caliper (**a**). The osteotomy was performed after temporary fixation with K-wire (**b**). Intraoperatively, the PSI was placed on the planned position with navigation pin and calipers contributed to precise aid positioning of the PSI (**a**). Each PSI was temporarily fixed with two or three K-wires. The saw blade was aligned with the PSI slope and a radial closed wedge osteotomy was performed with an oscillating saw (**b**).

Intraoperatively, the PSI was placed on the planned position with navigation pin and calipers, under c-arm fluoroscopy images that had contributed to the precise positioning of the PSI (Figure 3). Each PSI was temporarily fixed with two or three K-wires. The saw blade was aligned with the PSI slope, and a radial closed wedge osteotomy was performed with a Micro E oscillating saw (Rancho Cucamonga, CA, USA).

**Figure 4:**
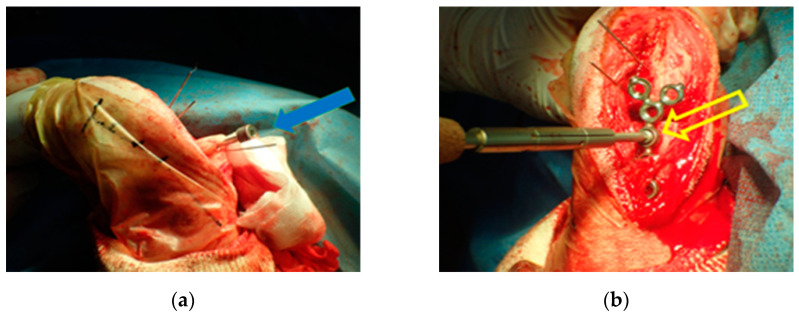
The proximal patient-specific instrumentation (PSI) unit has a design element for the drill sleeve hole (**a**). The pre-contoured locking plate (YULP) was first fixed to the proximal bone fragment on the cranial bone surface using a pre-drilled hole (**b**).

**Figure 5:**
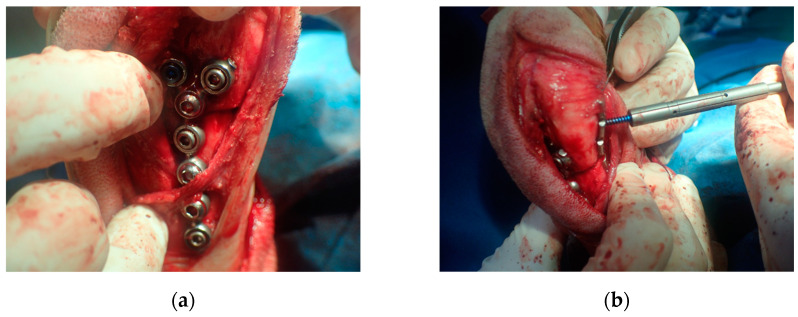
Locking plate (YULP) and locking screws on the cranial surface of the distal radius (**a**). The second locking plate and screws were placed on the caudomedial surface as planned (**b**).

**Figure 6:**
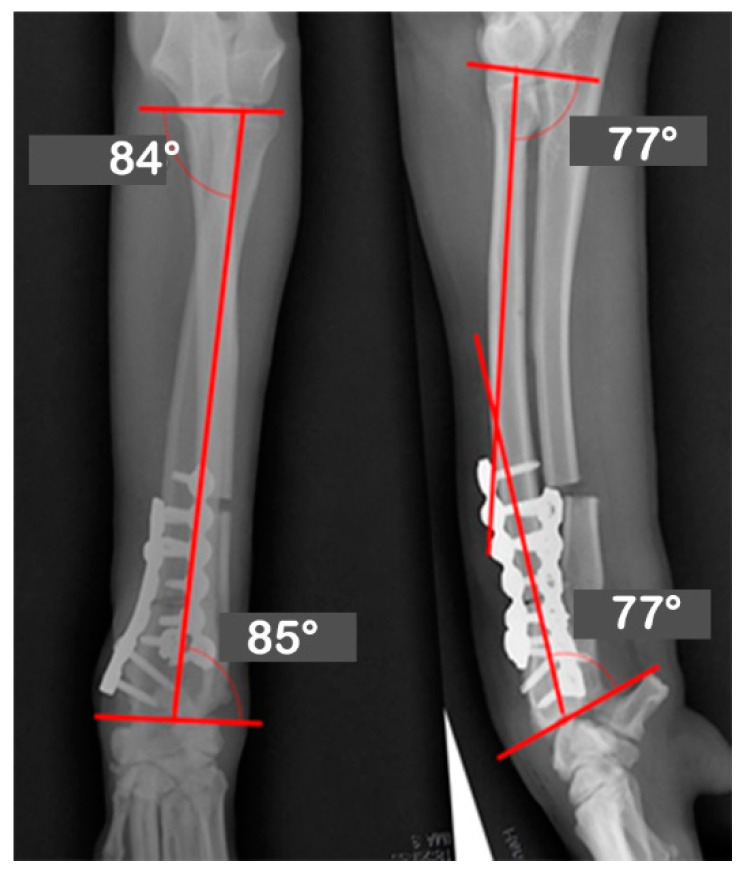
The immediate postoperative radiographs with the joint orientation line precisely meet the target angles as planned.

The osteotomies were performed, resulting in proximal and distal fragments. Next, a bone screw hole was predrilled through the SK ESF Drill Sleeves drill sleeve (IMEX Veterinary, Inc., Rancho Cucamonga, CA, USA) in the proximal PSI unit (Figure 4). The PSI was removed, and the carpus was aligned intraoperatively to the elbow to correct the external rotation. Each fragment had five locking screws and was connected by two plates. At least three locking screws were placed on each side of the bone fragment. The pre-contoured locking plate (YULP) was first fixed to the proximal bone fragment on the cranial bone surface using a pre-drilled hole, then the distal fragment was reduced directly by plate fixation. The second fixation was performed with a pre-contoured compression locking plate (CLP) on the medial bone surface as planned without any difficulty (Figure 5). In this surgery, we do not use or promote bone healing methods such as a cancellous bone graft. The surgical wound was closed in a routine manner. Orthogonal postoperative radiographs showed accurate implant positioning and antebrachial alignment (Figure 6).

### 2.6. Post-Operative Care

The dog recovered from anesthesia without complications, and carprofen (1.5 mg/kg, orally every 24 h for seven days) was administered for analgesia. Cage rest was directed for four weeks. The activity was restricted to a 10-min walk with a short lead walking for toileting twice per day and gradually increased until the fracture line disappeared.

## 3. Results

The dog was in excellent physical condition preoperatively. A general physical examination, thoracic radiographic evaluation, and laboratory investigations, including hematological, serum biochemical analysis, and urinalysis, were performed before the anesthesia, and all the results were within the normal limit. C-arm fluoroscopic intraoperative imaging was performed during the operation to confirm PSI positioning, K-wire pinning, and pre-drilling in situ. The time consumed for registration of the distal unit and proximal unit was 9 min 6 s and 1 min 20 s, respectively; for osteotomy of the distal and proximal cutting planes was 1 min 45 s and 51 s, respectively. No complications occurred during the anesthesia or immediately after surgery. The osteotomy results were confirmed as pre-operatively planned by orthogonal radiographic images. As shown in the preoperative surgical planning and simulation, the bone fixation implants could be mounted without interference between the screws of the two plates. The summary of the post-operative joint orientation angles evaluation for the corrective osteotomy is presented in Table 1 and Table 2. The reference values in Table 1 and Table 2 were collected from a previous report [24].

The surgical site edema reduced significantly on the second day after the operation, so the gentle pressure bandage for the surgical site edema was removed on the third day after the operation.

**Figure 7:**
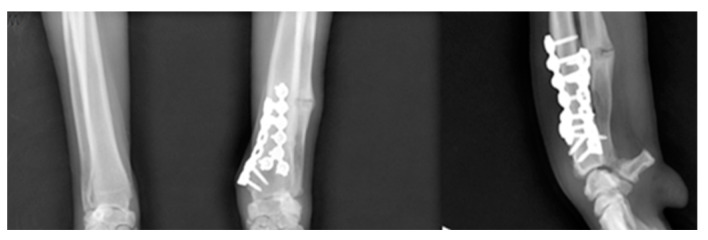
At the final 12-week postoperative radiography. The fracture-line on the distal radius had disappeared.

The patient improved significantly after two weeks. A lameness and mild painful carpal palpation with reduced carpal flexion range of motion (ROM) signified were noticed. Radiographs were rechecked immediately post-operatively and 12 weeks later. The radiographic findings showed excellent healing of the osteotomy. The 12-week radiographs showed osteogenesis with enhanced radiopacity in the radial callus formation (Figure 7). After 8 weeks, weight-bearing had improved, and the radiographs showed a progressive new bone formation at the osteotomy sites. At the final 12-week postoperative check, the patient presented no lameness with improved, pain-free carpal ROM, and increased antebrachial muscular tone on palpation.

## 4. Discussion

We describe a novel method for precise planning of a closing wedge osteotomy in 3-D using CT and CAD applications. RP modeling was used to create models for saw guide fabrication, bone plate pre-contouring, and rehearsal surgery. The patient had a successful outcome with significantly improved limb use and cosmetic appearance. Carpal degeneration may be reduced following the reform of the loading through the antebrachial bones.

Ideally, the veterinary surgeon will have access to the normal axis, as determined in 3-D, on the opposite limbs obtained from measurements of several individuals unaffected animals or with the same breed description. Currently, however, it is difficult to search for antebrachial joint orientational angle data on other breeds of dogs. Hence, for the work, we selected the Golden Retriever dog as the animal model, which had the same skeletal structure and normal value research data of joint orientation angles and lines [19].

Due to the deformity, there was a superimposition of the radiocarpal and accessory carpal bones with the distal radius and carpal rotation, which obscured the normal radiographic landmarks. It may have been possible to use the segmental radiography method outlined by Dismukes et al. [24] to avoid the superimposition of the carpal bones and the radius caused by the rotation. However, as this method is still two-dimensional, it was decided to use 3-D CT to augment and improve our understanding of the deformity within the radius.

The decision to use CT in the planning of this case stemmed from the need for more precise and accurate joint orientational angles to perform close wedge corrective osteotomy.

The CT image gave us not only the 3-D virtual visualization to try a range of potential osteotomies and assess the optimal site and angle of correction, but also real-sized antebrachial bone models and patient-specific cutting guides that can be successfully used for ALD. However, pre-operative labor does increase, especially computing work, which was significantly more when the 3-D printer was used [25,26]. CT allowed us to handle the deformity in 3-D bone models. In this way, CT enables rehearsal surgery to prevent potential mistakes in the operating room, and in the process, it can improve the protocol [27,28]. In our case, we were able to improve the protocol by finding out the problem with the osteotomy plan during the rehearsal surgery and assessing the risks of over- or under-correction as it is not possible to adjust during the postoperative period and revise the surgical plan.

Computer simulation has been described in human surgery and has been used for corrective osteotomies for the treatment of angular deformities, including malunions [20,29,30,31,32,33]. After reading reports about human surgery, we decided to create our simple cutting guides by molding PMMA onto the plastic model abutting our outlined cut planes. It improved the accuracy of the rehearsal and subsequent surgery. Rather than visually translating measurements from the CT data, the location and angle of the osteotomy were determined by the saw guides that were held rigidly in position on the patient’s radius.

It is reported in human surgery that the creation of a cutting guide template from a model and surgical simulation overcomes the inaccuracy associated with the translation of the rehearsal surgery to the patient. The accuracy of the osteotomy is determined by the resolution of the CT data (1 mm) and the accuracy of the model reproduction (0.18 mm), rather than the surgeon’s intraoperative measurements and judgment [32,33].

Our simple guides aided the use of the oscillating saw and illustrated how a specifically designed template or jig would be greatly advantageous. Stereo-lithographically produced jigs can be fabricated from plastic or metal as shells and used in the same manner as the PMMA guides used in this case. With greater familiarity with the technique, the surgery could be rehearsed electronically, and the saw guides themselves could be printed without the direct requirement for the bone models. The use of specifically manufactured surgical templates is an area that requires further study and may have application in veterinary surgery.

The major limitation of this study was that it was the first attempt at the proposed method. Both unfamiliarity with the capabilities of the software systems and the lack of normal data contributed to the flaws in the method that we reported. However, with further work, it will be easier to determine whether this method is primarily valid and how it relates to previously described methods of radiographic measurements.

The PSI design, in this case, did not require the modification of the angle to reduce the correction or leave a degree of cranial bowing. Although this adjustment was empirical as it was based on the already defined wedge, it was performed with confidence and the changes could be rehearsed.

The use of CT data to create stereolithographic models to aid in surgery is widely reported in the human literature, and there are early reports in the veterinary literature as well [24,34,35,36,37]. It has seen the most use in craniomaxillofacial surgery, but it is also used in many areas of orthopedics and, more recently, human cardiovascular surgery [38,39,40]. Models can be used to rehearse surgery, contour implants, improve diagnostic abilities, and aid in both the clinician’s and the involved client’s understanding. Human studies have concluded that the use of stereolithographic bio-models in combination with standard imaging has greater utility in surgical management than standard imaging data alone [38]. It is also reported that surgeons feel the use of bio-models reduces the time for surgical procedures; although, due to the inability to repeat surgery with the two methods, this observation is purely an anecdotal finding [36,38].

The positioning of the bone plate in situ is one of the vital issues for the outcome of computer-assisted osteotomies. The plate rigidly defined the displacement of the distal radius relative to the radial shaft. Thus, the surgeon had to ensure that the screw holes were drilled in the right locations and that the plate lay flat and perpendicular to the bone fragments. If these conditions were not met, errors in the placement of the plate and, hence, in the final position of the distal radius could occur. If the holes for the fixation screws were not drilled in the planned location, errors in translations and rotations could be induced in the location of the distal radius.

Of particular concern is the location of the most proximal hole of the proximal radial bone fragment: a literal translation of that hole by 1.0 mm can cause the second proximal hole to be a pivot point that rotates the plates and, hence, the centroid of the distal radius by approximately 1.9 mm. Since the bone surface is not planar, angular errors could be introduced. Traditionally, to attach the screws to the distal radius, the bone surface to which the fixation plate was to be attached needed to be shaved flat [41]. Failure to properly shape the bone could introduce translational errors in the y- and z-axes and rotational errors about x- and z-axes. However, using the locking screw plate in this method does not require the shaving procedure, which potentially creates an error [42]. The radial shaft is approximately cylindrical. This geometry allows for small rotational errors about the shaft’s axis to pass undetected. In addition, the screw holes in the fixation plate allowed the screw to lay anywhere within a cortical region. This reduced the effectiveness of the screw as a spatial constraint of the plate–bone system [43]. However, locking screw and plate usage can solve this problem. For the screws, pilot holes were created using K-wires of 1.2 mm diameter. For each such wire, the drill had to be calibrated, introducing a possible source of error. Due to the length of the wire protruding approximately 50 mm from the drill chuck and the weight of the drill, bending of the wire during the procedure was possible. If such bending occurred during the calibration registration or alignment of the wire to the drill locations, an error could be introduced in positioning the drill location and/or orientation. Careful calibration and use of the drill can reduce such errors. Though this was not strictly part of the planning, it is vital to mention its importance for the overall outcome of a 3-D computer-aid procedure. Registration was used to find the transformation from the proximal target coordinates to the CT coordinates [29]. A poor registration could have led to a misplaced drill location and, hence, a misplaced fixation plate. Robust algorithms, including the contouring on the real-size RP bone model and validation of the registration, can reduce the likelihood of poor registration.

Although this study, a 3-D-based assessment, can be more useful for complex deformities [44,45], the post-operative evaluation relied on 2-D radiographs.

We found that having the models was very valuable in the planning of the case, not only for the procedure itself but also for explaining the procedure to the owner. This case report showed the application of this new technology in veterinary surgery and demonstrated that it is possible to improve the accuracy of surgical procedures involving complex geometry. It does, however, highlight the need for further study. Furthermore, an assessment of the extra cost of CT and 3-D printing work, and a comparison of the analysis of merits and demerits achieved using traditional radiography versus 3-D-aided surgical protocol is needed.

## 5. Conclusions

From this in-house surgical procedure, we concluded that the advanced data gained with digital imaging, stereolithographic models, 3-D printed real-sized bone models, and fabricating PSCGs may benefit the patient, client, and surgical team. This protocol enriched the morphological experience of bone deformities, aided client communication, gained the surgeon’s confidence, maximized surgical precision, minimized surgical wound exposure and anesthetic times, and was intuitive. We propose to veterinary surgeons that computer-aided orthopedic surgery affords many advantages and advocates further research.

## Figures and Tables

**Table 1:** Joint orientation angles with joint orientation lines.

Golden Retrievers		Mean Value	Reference Value
Frontal	aMPRA	83	82~83
	aLDRA	86	85~87
Sagittal	aCdPRA	85	84~86
	aCdDRA	77	76~78
	Θ	27	21~32

aMPRA, anatomical medial proximal radial angle; aLDRA, anatomical lateral distal radial angle; aCdPRA, anatomical caudal proximal radial angle; aCdDRA, anatomical caudal distal radial angle; Θ, the angular intersection (procurvature).

**Table 2:** Joint orientation angles before and after surgery.

JOA	Pre-OP	Post-OP	Target	Mean	Reference
				Values	Values
aLDRA	57	85	85	86	85~87
aCdPRA	92	87	87	85	84~86
aMPRA	75	84	84	83	82~83
aCdDRA	62	77	77	77	76~78
θ	38	21	21	27	21~32

Joint Orientation Angles (JOA) of pre-operative (Pre-OP), post-operative (Post-OP), Target, and mean values aMPRA, anatomical medial proximal radial angle; aLDRA, anatomical lateral distal radial angle; aCdPRA, anatomical caudal proximal radial angle; aCdDRA, anatomical caudal distal radial angle; θ, Angular intersection of two segmental joint reference axes.

## Data Availability

Not applicable.
